# Callus Formation in Fractured Femur of Rats Treated with Injection of Human Umbilical Cord Mesenchymal Stem Cell-Conditioned Medium

**DOI:** 10.1155/2021/8410175

**Published:** 2021-04-27

**Authors:** Marla Anggita, Widagdo Sri Nugroho, Yuda Heru Fibrianto, Setyo Budhi, Teguh Budipitojo

**Affiliations:** ^1^Postgraduate Student of Veterinary Science Master Program, Universitas Gadjah Mada, Yogyakarta 55281, Indonesia; ^2^Department of Veterinary Public Health, Faculty of Veterinary Medicine, Universitas Gadjah Mada, Yogyakarta 55281, Indonesia; ^3^Department of Physiology, Faculty of Veterinary Medicine, Universitas Gadjah Mada, Yogyakarta 55281, Indonesia; ^4^Department of Surgery and Radiology, Faculty of Veterinary Medicine, Universitas Gadjah Mada, Yogyakarta 55281, Indonesia; ^5^Department of Anatomy, Faculty of Veterinary Medicine, Universitas Gadjah Mada, Yogyakarta 55281, Indonesia

## Abstract

Mesenchymal stem cells-conditioned medium (MSC-CM) is the extraction from stem cell medium containing biological substances, including growth factors and cytokines. These substances play roles in the various functions of body regulatory, including bone formation. However, the effect of MSC-CM derived from human umbilical cord injection in femur fracture healing of rats has not been reported previously. This study aims to see the effect of MSC-CM derived from human umbilical cord injection on the callus formation of bone fracture healing in Wistar rats (*Rattus norvegicus*). A femur fracture in 54 Wistar rats was made by surgery according to the procedure under sterile conditions. After the surgery, rats were divided into 2 groups of 27, respectively. Injection in the control (0.1 mL/kg body weight NaCl) and MSC-CM group (0.1 mL/kg body weight MSC-CM) was performed on weeks 0, 1, 2, 3, 4, 5, 6, 7, and 8 after surgery. Radiographic images and the femur bone samples were taken and collected on days 1, 7, 14, 21, 28, 35, and 60 after surgery. Bone samples were then fixed in Bouin solution. Histologic preparations were done by the paraffin method, by cutting the tissue blocks into 5 *μ*m thickness and then staining with Mallory aniline blue staining. The results were analyzed descriptively and quantitatively. The result showed that the soft callus formation occurred rapidly and got wider in the MSC-CM group than that of the control group. The administration of MSC-CM injection postfracture surgery to femur fracture cases in rats was capable to accelerate the callus formation.

## 1. Introduction

Mesenchymal stem cell (MSC) is a pluripotent cell that can be differentiated into many kinds of a cell, such as a chondrocyte, osteocyte, adipocyte, myocyte, and neuron [1]. Mesenchymal stem cell can be isolated from adipose tissue, bone marrow, placenta, umbilical cord, olfactory mucous, deciduous teeth, lien, brain [2], blood cell, amnion, vein, Wharton's jelly, and umbilical cord matrix cell [3]. In the application, MSC can differentiate a mature cell and fill the damaged tissue, secreted cytokine, and other soluble mediates for tissue regeneration and mediator for protein release [4]. Stem cell has been used in many studies and gave a good result in the treatment of degenerative diseases, such as myocardial infarction [5], diabetes [6], bone marrow failure [7], Parkinson's disease [8], and liver cirrhosis [9].

In most cases of stem cell application, there is no convincing evidence against the statement that stem cells could rebuild damaged tissue after administration. The therapeutic effect of stem cell arises from their secreted factors, such as growth factor, cytokine, chemokine, and metabolites, which acts as a biologic regulator in the autocrine and paracrine body function [10]. In line with the previous statement, there were some findings to reveal that factors secreted by MSC have therapeutic effects for antiapoptosis, angiogenic, anti-injury, immunomodulatory, and chemoattractive activity [11] and increase neuronal growth and durability [2].

The administration of a biological substance involved in the healing process of the bone can accelerate the fracture healing time. Various biological substances have been reported in accelerating bone healing. The administration of bone morphogenesis proteins [12], recombinant TGF-*β* [13, 14], recombinant human basic fibroblast growth factor (hbFGF) [15], and human parathyroid hormone [16] gave a good result in fracture healing, as shown by the acceleration of callus growth in the early phase of the bone healing process.

This study aims to clarify the effect of injection of human umbilical cord mesenchymal stem cells-conditioned medium on the callus formation of fracture healing in rats.

## 2. Materials and Methods

### 2.1. Ethical Approval

The experimental animals used in this study were approved by the Ethical Clearance Committee from Universitas Gadjah Mada, Indonesia (reference number: 00056/04/LPPT/X/2016).

### 2.2. Animal Model

Fifty-four 3-months old male Wistar rats (*Rattus norvegicus*), 200–250 g in weight, were used in this study. Rats were adapted for 7 days and fed with ad libitum. Fracture-making surgery was performed on day 8. After conducting femur fracture-making surgery in all rats, we divided them into 2 groups: control and MSC-CM group. Animals in the control group received 0.1 mL/kg b.w. NaCl intramuscular injection, and MSC-CM group was given 0.1 mL/kg b.w. MSC-CM intramuscular injection was performed once a week at weeks 0, 1, 2, 3, 4, 5, 6, 7, and 8 after surgery. The observation period lasted for 2 months.

### 2.3. Human Umbilical Cord Mesenchymal Stem Cell-Conditioned Medium

Through 4 passages, the culture of MSCs derived from the human umbilical cord that has reached 60% confluence was harvested with a warm trypsinization model. After the neutralization of trypsin, the cell suspension was centrifuged at 3000 rpm for 10 minutes. The supernatant was removed, and the cell deposits were washed with PBS 3 times. Cell deposits were then resuspended with a new medium with a concentration of 10,000 cells per mL. The stem cells were modified into an embryoid body and planted on a culture medium with a complete medium until confluence was formed between embryoid bodies. MSC-CM production was carried out by washing embryoid body cultures with sterile PBS and filling the embryoid body culture plate with 10 mL of complete medium without serum. After 48 h, the MSC-CM was stored at −20°C until used.

### 2.4. Surgical Procedure

Right femoral fracture in rats was made by surgery according to the standard operating procedure under sterile conditions. Combination of 10% ketamine (ketamine 10% inj., PT. Otasindo Prima Satwa, Indonesia) and 2% xylazine (Xyla, PT. Tekad Mandiri Citra, Indonesia) with a dose rate of 75 and 5 mg/kg^−1^ body weight, respectively, were used for rats anesthetized [17]. Femoral osteotomies were performed with a bone saw. Intramedullary pen (18G needle, Terumo®) was mounted on the fracture site as a fixative. After the surgery, ampicillin (ampicillin sodium for injection, Tianjin Glory Technology Co., Ltd., China) 20 mg/kg b.w. [18] was given via intramuscular injection once a day for 5 days as an antibiotic.

### 2.5. Sample Processing and Data Analysis

Radiograph images were taken at days 1, 7, 14, 21, 28, 35, and 60 after surgery. Bone samples were collected at days 1, 7, 14, 21, 28, 35, and 60. One rat from each group was euthanized, and then the right femoral bone was collected. The bone samples were then fixated in Bouin's solution. Bone samples were decalcified in the solution of ethylenediaminetetraacetic acid (EDTA) 10% for 3 weeks. Histologic preparations were done by the paraffin method by cutting the tissue blocks into 5 *μ*m thickness and then stained with Mallory aniline blue staining. The results were being analyzed descriptively and quantitatively. The callus area was measured using Image Raster v.3. software and statistically analysed using ANOVA with *p* < 0.05.

## 3. Results

### 3.1. Radiographic Examination

**Figure 1:**
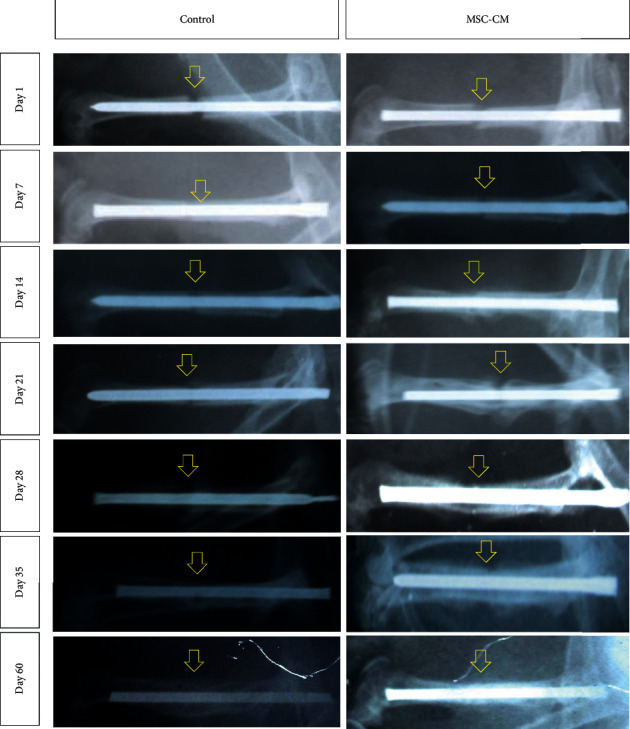
Radiographic images of rats femur bone in control and MSC-CM groups on days 1–60 after surgery. The yellow arrow indicates the gap fracture area of the bone. The callus formed around the gap fracture area is more radiolucent than the compact bone.

We observed the gap fracture and callus formation on days 1, 7, 14, 21, 28, 35, and 60 after surgery. On radiographic examination, the callus appears to be greyish (more radiolucent) than compact bone (more radiopaque). Radiographic images showed the gap fracture in both groups on days 1–7 after surgery (Figure 1). The callus was formed on day 14 after surgery in the MSC-CM group, while in the control group, it was formed on day 28. The callus continuously formed, and at day 60, the callus had finally formed into compact bone (no difference in color between callus and compact bone, considered as compact bone) in both groups.

### 3.2. Histological Observation

Mallory aniline blue (MAB) staining was carried out to determine the area of soft and hard callus in the femur bone tissue. We measured the callus area of both groups from day 1 until day 60 after surgery. Callus areas were measured to determine the speed of the healing process achieved in both groups. Soft callus consists of cartilage tissue and colored light blue in MAB staining, and the hard callus consists of lamellar bone matrix colored dark blue in MAB staining. The callus area was measured by Image Raster v.3. software. The result is presented in Table 1.

The ANOVA test result showed that there was no significant different between the soft callus formation in both groups (*p* < 0.05), but the hard callus formation showed significant differences in control and MSC-CM groups in days 35 and 60 after surgery (*p* < 0.05).

**Figure 2:**
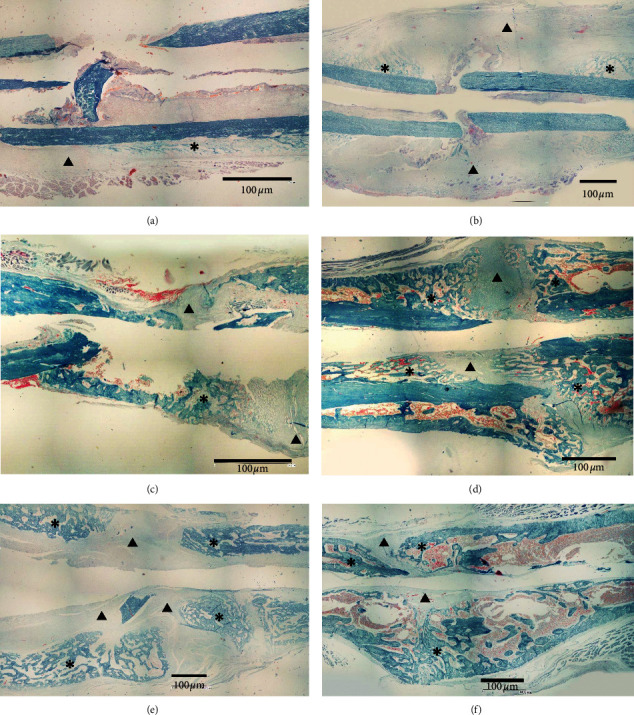
Histologic images of fracture bone tissue of rats on day 7, 35, and 60 after surgery. On day 7 after the surgery, the soft callus area (triangle) in the control group (a) was narrow than the MSC-CM group (b). On day 35 after the surgery, the soft callus area is still wider on the control group (c), while in the MSC-CM group (d), the hard callus area was dominant. On day 60 after the surgery, in the control group (e), the soft callus area still dominated the bone tissue, while the soft callus area was almost entirely replaced by the hard callus area (star) in the MSC-CM group (f).

Callus area was measured from day 1 after surgery until day 60. The soft callus area containing proliferative and hypertrophic chondrocytes is interpreted in light blue, and the hard callus area containing the lamellar bone matrix is interpreted in dark blue in MAB staining (Figure 2). The formation of soft callus in both groups started at day 7. Soft callus and hard callus continued to form at day 21–28. At day 60 after fracture, the hard callus area in the MSC-CM group was wider than that in the control group. However, MSC-CM group was dominated by hard callus, while in the control group, the soft callus remained.

In this study, we measured the callus formation that occurred in the fractured femur of rats in both groups through the observation of radiographic results and the formation of soft and hard callus through the Mallory aniline blue staining. Based on radiograph images, histological images, and callus measurements, it was found that the growth rate of callus in MSC-CM group rats was achieved faster than rats in the control group.

## 4. Discussion

On day 7 after the surgery, the soft callus in the control group only appeared on one side of the bone's fragment, but the MSC-CM group showed a different result, where the callus was already connected the bone's fragments and made a bridge across them. However, based on the radiographic image, the callus growth of two groups was visible on day 14 after the surgery, where the MSC-CM groups showed the more extensive growth of callus than that of the control group. Mesenchymal stem cells-conditioned medium derived from human umbilical cord has been previously reported by Kim et al. [19] which contained endothelial growth factor (EGF), vascular endothelial growth factor (VEGF), granulocyte colony stimulating factor (GCSF), granulocyte macrophage CSF (GM-CSF), transforming growth factor-*β*1 (TGF-*β*1), platelet-derived growth factor (PDGF), basic fibroblast growth factor (bFGF), and keratinocyte growth factor (KGF). Platelet-derived growth factor, VEFG, bFGF, IL-6, KGF, and TGF-*β* play an important role in fracture repairs, such as cell proliferation, differentiation, and homeostasis [20]. Transforming growth factor-*β*2, -*β*3, PDGF, and FGF stimulated the proliferation of fibroblasts and chondrocytes [21]. Chondrocytes responded to all of these factors by secreting extracellular matrix protein, such as collagen II [22]. Thus, the extracellular matrix and collagen secreted by the chondrocytes made the callus growth faster and finally be able to make a bridge between the fractured bones.

Fracture healing is a complex process and involves the role of the various cells, growth factors, and extracellular matrix of the bone [23]. In fracture healing, there was an occurrence of the secondary bone healing (indirect bone healing). This process includes inflammation or hematoma (24–72 h), repair or callus formation (occurs 1-2 weeks after fracture), and remodeling [24–26]. At day 60 after the surgery, the radiograph image showed that the callus was already connected into compact bone, with the histologic images showing the same result, where the soft callus was already replaced with the hard callus. The hard callus contains the combination of high protein and mineralization extracellular matrix. This matrix especially was synthesized by mature osteoblasts. To form hard callus, the soft callus tissue should be removed. The end phase of fracture healing is the change of hard callus/woven bone into cortical bone. Interaction and signaling of various factors involved in the fracture healing process are important for optimal repair of the bone. The replacement process of soft callus to hard callus can be viewed from the result of callus measurement. The formation of hard callus indicated the end phase of fracture healing, and the bone would then go through the remodeling phase.

Various studies have been conducted to see the effect of MSC-CM on bone regeneration, and among others are the use of MSC-CM from adipose cells for bone regeneration [27], MSC-CM from human mesenchymal stem cells for alveolar bone regeneration [28], and MSC-CM derived from human fetal tissue for distraction osteogenesis [29]. Overall, the administration of MSC-CM to bone fracture cases gives good results as indicated by the accelerated regeneration of the bone. The extracts of MSCs growth media used in this study have also been reported to give good results on the regeneration of skin burns [30], skin wound [31], and beta cells of the pancreas in rats with type 1 diabetes mellitus [32].

In older patients or patients with bone disorder, the administration of biological substances that support the growth of the mesenchymal stem cell of the bone will affect the results of bone recovery [33]. However, not much is known about the mechanism of healing that occurs in the damaged tissues after the local administration of MSC-CM. It is unclear whether the factors contained in MSC-CM induced the cells proliferation and differentiation in the damaged tissues or the factors contained in MSC-CM induced the cells in the damaged tissues to produce other factors that are needed to regenerate the tissues in the body. Thus, we need to conduct further study to have a complete understanding regarding the way the MSC-CM could accelerate the regeneration of the damaged tissues with the aim of finding an effective way for implementing the MSC-CM in degenerative disease cases. It is also necessary to know in detail the exact content in the MSC-CM derived from the human umbilical cord used in this study.

## 5. Conclusions

It was found that the administration of MSC-CM in cases of fractures did not have any significant different on the acceleration of bone healing process. However, the administration of MSC-CM significantly affected the increase of the hard callus growth on days 35 and 60 after surgery. There is a possibility that the factors contained in MSC-CM, such as TGF-*β*, PDGF, and FGF, are involved in the process of proliferation and differentiation of mesenchymal cells of the bone, thus leading to the hard callus formation in the MSC-CM group than in the control group.

## Figures and Tables

**Table 1:** Callus area measurement (mm^2^).

Day ^*∗*^	Soft callus		Hard callus	
	Control	MSC-CM	Control	MSC-CM
1	0	0	0	0
7	3.5	23.6	2.1	4.6
14	6.2	7.8	4.4	9.6
21	4.5	6.3	4.1	10.7
28	2.4	4	2.5	11.5
35	22.7	6.7	10^b^	11.8^a^
60	21	2.4	15.8^c^	16.1^c^

^*∗*^Days after fracture surgery. ^a, b, c^Significantly different (*p* < 0.05).

## Data Availability

The data used in this study are available from the authors upon request.
